# Version Reporting and Assessment Approaches for New and Updated Activity and Heart Rate Monitors †

**DOI:** 10.3390/s19071705

**Published:** 2019-04-10

**Authors:** Tim Collins, Sandra I. Woolley, Salome Oniani, Ivan Miguel Pires, Nuno M. Garcia, Sean J. Ledger, Anand Pandyan

**Affiliations:** 1 School of Engineering, Manchester Metropolitan University, Manchester M15 6BH, UK; 2 School of Computing and Mathematics, Keele University, Staffordshire ST5 5BG, UK; s.i.woolley@keele.ac.uk; 3 Faculty of Informatics and Control Systems, Georgian Technical University, Tbilisi 380075, Georgia; s.oniani@gtu.ge; 4 Instituto de Telecomunicações, Universidade da Beira Interior, 6201-001 Covilhã, Portugal; impires@it.ubi.pt (I.M.P.); ngarcia@di.ubi.pt (N.M.G.); 5 Altranportugal, S.A., 1990-096 Lisbon, Portugal; 6 ALLab—Assisted Living Computing and Telecommunications Laboratory, Computing Science Department, Universidade da Beira Interior, 6201-001 Covilhã, Portugal; 7 ECATI, Universidade Lusófona de Humanidades e Tecnologias, 1749-024 Lisbon, Portugal; 8 School of Health and Rehabilitation, Keele University, Staffordshire ST5 5BG, UK; s.j.ledger@keele.ac.uk (S.J.L.); a.d.pandyan@keele.ac.uk (A.P.)

**Keywords:** wearable sensing, activity monitoring, ambulatory heart rate, inter-instrument reliability

## Abstract

This paper addresses the significant need for improvements in device version reporting and practice across the academic and technical activity monitoring literature, and it recommends assessments for new and updated consumer sensing devices. Reproducibility and data veracity are central to good scholarship, and particularly significant in clinical and health applications. Across the literature there is an absence of device version reporting and a failure to recognize that device validity is not maintained when firmware and software updates can, and do, change device performance and parameter estimation. In this paper, we propose the use of tractable methods to assess devices *at their current version* and provide an example empirical approach. Experimental results for heart rate and step count acquisitions during walking and everyday living activities from Garmin Vivosmart 3 (v4.10) wristband monitors are presented and analyzed, and the reliability issues of optically-acquired heart rates, especially during periods of activity, are demonstrated and discussed. In conclusion, the paper recommends the empirical assessment of new and updated activity monitors and improvements in device version reporting across the academic and technical literature.

## 1. Introduction

Consumer wearable monitoring devices are used across a spectrum of health, well-being and behavioral studies, as well as clinical trials. For example, the U.S. Library of Medicine ClinicalTrials.gov database reports over 240 “Completed” to “Not yet recruiting” trials involving Fitbit devices (search accessed 3 January 2019). However, the manufacturers of these devices are generally very clear that activity trackers are not medical devices. For example, Garmin Vivosmart “Important Safety and Product Information” [1] advises that the device is for “*recreational purposes and not for medical purposes*” and that “*inherent limitations*” may “*cause some heart rate readings to be inaccurate*”, similarly, Fitbit device “Important Safety and Product Information” declares that the device is “*not a medical device*” and “*accuracy of Fitbit devices is not intended to match medical devices or scientific measurement devices*” [2]. However, given that these devices *are* being used in clinical applications, and with future clinical applications anticipated [3], their validity and reliability are important.

In terms of meeting expectations, it is noteworthy that, at the time of writing, Fitbit’s motion to dismiss a class action was recently denied. The complaint alleged “*gross inaccuracies and recording failures*” [4] because “*products frequently fail to record any heart rate at all or provide highly inaccurate readings, with discrepancies of up to 75 bpm*” [5].

This invited paper is an expansion of the work presented in [6] which proposed reliability assessments of new and updated consumer-grade activity and heart rate monitors using Garmin Vivosmart 3 activity trackers as exemplar devices to illustrate an assessment approach. This paper is extended by the addition of step count results and Bland-Altman plots of heart rate acquisitions, and, also, in the following sections with accounts of underlying reliability issues relevant to ambulatory photoplethysmography and step counting.

### 1.1. Photoplethysmography (PPG)

**Figure 1:**
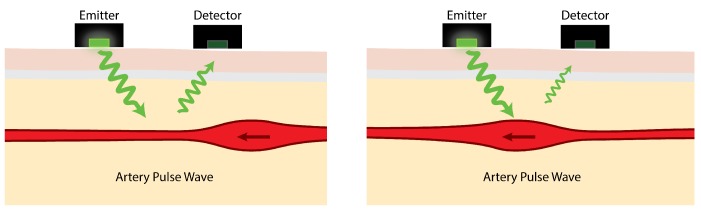
Light reflectance in photoplethysmography (PPG) [8].

Ambulatory optical heart rate acquisition from photoplethysmography (PPG) sensors is known to be very challenging [7]. As illustrated in Figure 1, light emitted by PPG sensors is mostly absorbed by body tissues. The amount of light reflected depends on several factors, one of which is the volume of arteries near the skin’s surface. Blood in the arteries and arterioles absorbs light better than the surrounding body tissues so, as they contract and swell in response to pulsating blood pressure, the intensity of reflected light rises and falls.

The reflected light variation due to arterial pulse waves is typically, at best, about 2% [9]. PPG sensors detect this small variation in reflected light and use it to estimate heart rate. The effects of movement at the sensor-skin interface can mean that simply walking can be enough to mask the pulse signal. Indeed, one of the main challenges is the range of severe interference effects caused by movement [10,11].

There are contrasting reports in the academic literature regarding consumer PPG heart rate monitor validity; some studies conclude devices are valid [12], whilst others report on systematic errors [13,14] or a scarcity of validation evidence [15], or recognize the multi-factorial nature of device performance [16]. One option to reduce PPG sensitivity to movement is to adhere the sensor over the carotid artery, but this is both intrusive and uncomfortable [17]. In addition to movement, optical heart rate signals can also be affected by skin color [18] and aging [19]. Yet, optical heart rate acquisition remains a desirable alternative to sensors such as electrocardiogram (ECG) chest straps for consumer-level activity monitors, where comfortability, ease-of-use and low cost are prioritized. It is, therefore, desirable that improvements in PPG heart rate estimation accuracy can be achieved in further research.

### 1.2. Step Counting

Step counting is a common function of consumer activity trackers. Step counts are estimated by analyses of accelerometer data which is filtered to attempt to isolate features caused by steps from those caused by other activities. Despite this filtering, erroneously logged steps can be produced by non-step activities such as folding laundry [20] and other tasks and actions [21], particularly when devices are worn on the dominant wrist. In addition to activity-induced false-positive step count errors, false-negative step count errors are also reported in the literature when, for example, monitors are used at slow walking speeds [21,22,23].

### 1.3. Device Selection, Assessment and Iteration

The selection of an appropriate activity monitor for a given study is typically determined by the required parameter acquisitions and deployment needs [24] as well as the study budget. However, the calibration and validation of devices [25,26] can be onerous. Best practice requires a substantial time and resource investment for researchers to calibrate and validate sufficiently large numbers of their devices with a large and diverse cohort of representative users performing a range of anticipated activities. At the same time, commercial monitors can frequently and automatically update both software and firmware that can alter device function, data collection and data reporting, all of which have the potential to compromise previous validations. However, of course, manufacturers are under no obligation to report the detail of their proprietary algorithms or the specifics of version changes.

Devices that have the same model name, but operate with different software and firmware versions, are distinct devices; they should not be treated as identical devices. Ideally, devices would be clearly differentiated in the literature with manufacturer, model and version data. While there may be limited (if any) opportunity for researchers to reversion commercial device software to repeat published experiments, the provision of version information would, at least, limit the potential for incorrect aggregations of data for devices that operate with different software and firmware versions. Unfortunately, there is a lack of studies in the literature comparing the performance of identical devices using different software and/or firmware versions.

A number of studies have assessed and compared the validity and accuracy of different monitoring device models [27,28,29]. However, across this literature, and in reviews of this literature [30], it is common practice to provide version data for the software used for statistical analyses of device performance, but it is not common practice to report version information for the devices themselves. As an example of device ambiguity, a reference to “Garmin Vivosmart” could refer to Garmin Vivosmart 4, Garmin Vivosmart 3 or Garmin Vivosmart HR. The date of a given publication might help disambiguate the model variant but will not help identify the version. The Vivosmart HR had 14 versions from 2.10 to 4.30 over approximately 30 months (each update comprising between 1 and 11 items, such as, “*improved calculation of Intensity Minutes*” and “*Various other improvements*”) [31]. At the time of the experimental work presented in this paper (May 2018), the Garmin Vivosmart 3 (v4.10) was the latest of nine versions and at the submission of this paper (January 2019) there had been a further six updates and the release of a new Vivosmart 4 device which itself has received four updates comprising 25 items.

The U.S. Food and Drug Administration has established a new ‘Digital Health Software Precertification (Pre-Cert) Program’ [32] that aspires toward a more agile approach to digital health technology regulation that recognizes the “*iterative characteristics*” of new consumer devices [33]. Ideally, study implementations of activity monitors would assess devices at their current ‘iteration’ and maintain devices at that version throughout the study. If this were not desirable or practicable, it would be preferable for update schedules to be controlled such that acquired data could be differentiated accordingly.

Given that device performance and functionality can change with version updates and that these updates can occur at frequent intervals, a tractable approach for assessing or reassessing devices is desirable. In the following section we present an exemplar assessment for a new or updated device. The assessment approach exemplified here is not, and could not be, prescriptive. A useful approach must incorporate participants and activities that have relevance to the intended study. It should also be emphasized that a comprehensive validity and reliability assessment using calibration devices would be preferable to the approach outlined here, but, the proposed sample-based approach is preferable to no assessment at all.

## 2. Method and Materials

**Figure 2:**
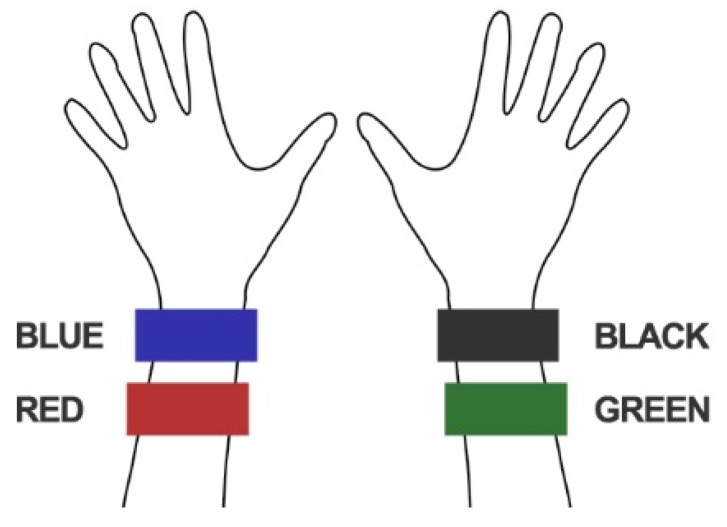
Activity monitor positions (color-coded for reference).

Four Garmin Vivosmart 3 activity trackers (all versioned SW v4.10 throughout data acquisitions during May 2018) were worn, as shown in Figure 2, by four healthy researcher participants, P01-P04 outlined in Table 1, during (i) the treadmill walking activities summarized in Table 2 and (ii) 12 h of everyday living. Two activity trackers were worn on each arm, both within the manufacturers recommended region of the wrist. The walking speeds: slow, moderate, fast and vigorous, were selected based on reports in the literature [34,35] and were performed on an h/p/cosmos Pulsar treadmill. To support reproducibility [36], we report further details about materials in Appendix A.

In many activity monitoring experiments, especially where devices are reused by participants, four devices will represent a sizable proportion of the total number of study devices and could constitute a worthy lot sample. In larger scale experiments and studies where substantial numbers of devices are to be deployed, and where more researcher time is available, larger test samples of devices would be appropriate.

All participants reported regularly partaking brisk-intensive exercise outside largely sedentary academic/working roles. Participant 1 was ambidextrous; all other participants were right-handed. (Ethical approval for “Health Technology Assessment and Data Analytics”, ERP2329 was obtained from Keele University and all participants gave their informed consent to take part.)

Walking speeds were regulated by the researcher programming the treadmill speed as per the schedule in Table 2. The slow walking activity was prefaced by two minutes of standing with arms down. Pulse readings were taken from a Polar H10 chest strap ECG monitor at 1-min intervals throughout the activity (one sample per minute is the fastest rate that the Vivosmart 3 devices log heart rate data; a shorter interval would be more appropriate if assessing devices with a higher sampling rate). Data (from logged Garmin .FIT files) was downloaded from the activity trackers after each activity and converted into .CSV files and imported into Excel. Dates and times were converted from the Garmin 16- and 32-bit timestamps used in the .FIT file [37] into standard Excel date-time serial numbers. Mean Absolute Percentage Error (MAPE), the IntraClass Correlation (ICC) and Bland-Altman plots, as commonly used in research reported in the literature [28,38], were used to compare the heart rate recordings from the activity trackers with the ECG chest strap reference. Step counts were also acquired from the trackers and between-device comparisons were made. Two-way, mixed, single measures ICC variants for assessing absolute agreement and for assessing consistency, as defined by McGraw and Wong [39], were applied.

## 3. Results

**Figure 3:**
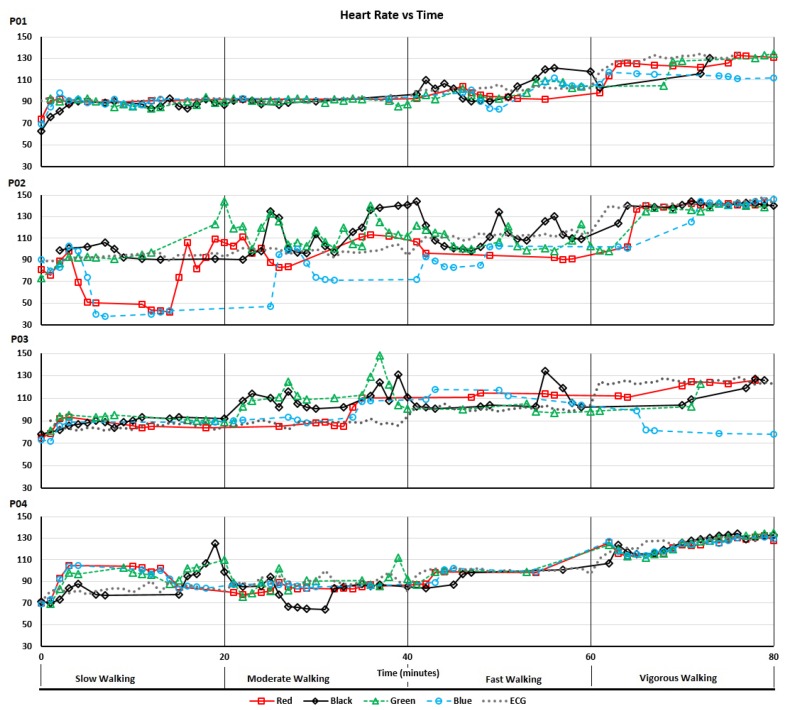
Heart rate recordings acquired during treadmill walking activities.

**Figure 4:**
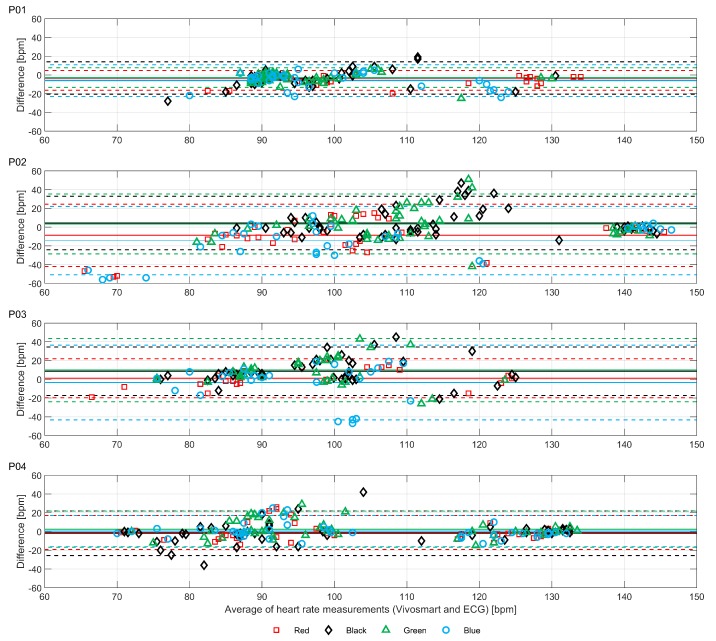
Bland-Altman plots for each device compared with electrocardiogram (ECG) chest strap for treadmill activities. Means (solid lines) and ±1.96SD levels (dashed lines) for each device are indicated.

Figure 3 shows the heart rate recordings for P01-P04 from the treadmill walking activities. Variability in recorded values can be seen at both slower and faster walking speeds and, notably, differs between participants. For analysis of the acquired data we calculated the MAPE (compared with the ECG chest strap reference) and ICC values listed in Table 3. As shown, treadmill acquisitions for participants P02 and P03 produced higher MAPEs (including MAPEs over 10%: the level often taken as the upper bound for “acceptable” errors) and lower ICCs. This could, in part, be attributed to the increased age of participants P02 and P03 compared to P01 and P04. As shown in Figure 3, for P02 there were some abnormally low but sparse heart rate recordings from the “blue” device and, to a lesser extent, the “red” device. For P03, the “blue” device recorded decreasing heart rates when the actual heart rate increased during the vigorous walking activity. This produced a near zero ICC. Bland-Altman plots of the same data are shown in Figure 4.

The Bland-Altman plots illustrate the substantial range of differences in heart rate estimation between the devices and the ECG chest strap reference. As shown in Figure 4, the largest differences do not occur at the highest average heart rates, but instead occur between approximately 90 and 120 bpm. The diagonal clustering of points within this range is consistent with systematic errors caused by interference from motion artifacts correlated with the walking rates of the participants (the average step counts per minute of the participants were 106, 105, 102 and 115, respectively for P01-04). Thus, the results shown in Figure 3 and Figure 4 demonstrate how, even with small sample sizes of devices and participants, the potential extent of erroneous readings is quickly revealed (i.e., large effects are visible in small sample sizes; small effects are only revealed with large samples).

**Figure 5:**
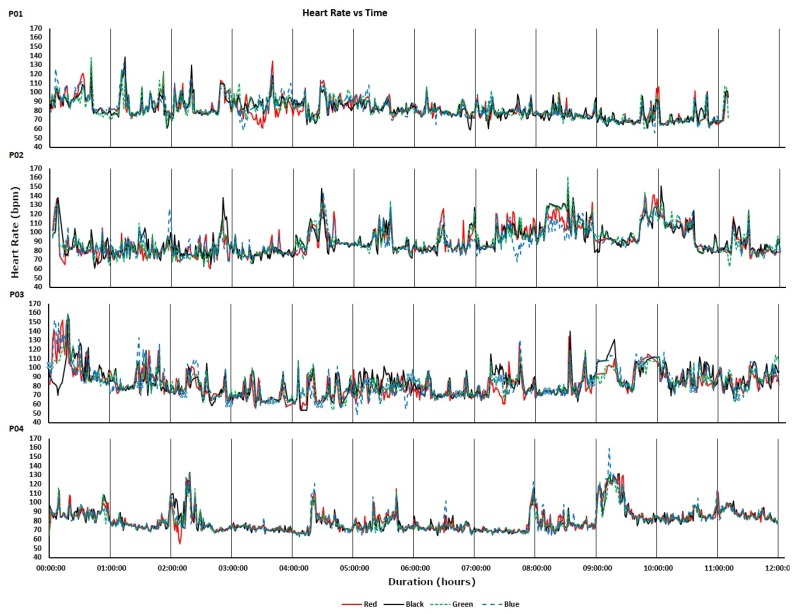
Heart rate recordings acquired during 12 h of everyday living.

**Figure 6:**
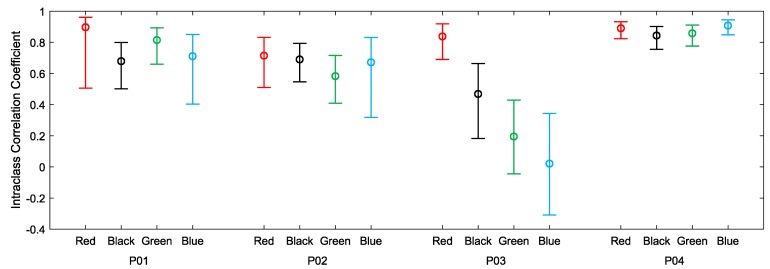
ICC for each device compared with ECG chest strap reference recordings with 90% confidence intervals for treadmill activities.

**Figure 7:**
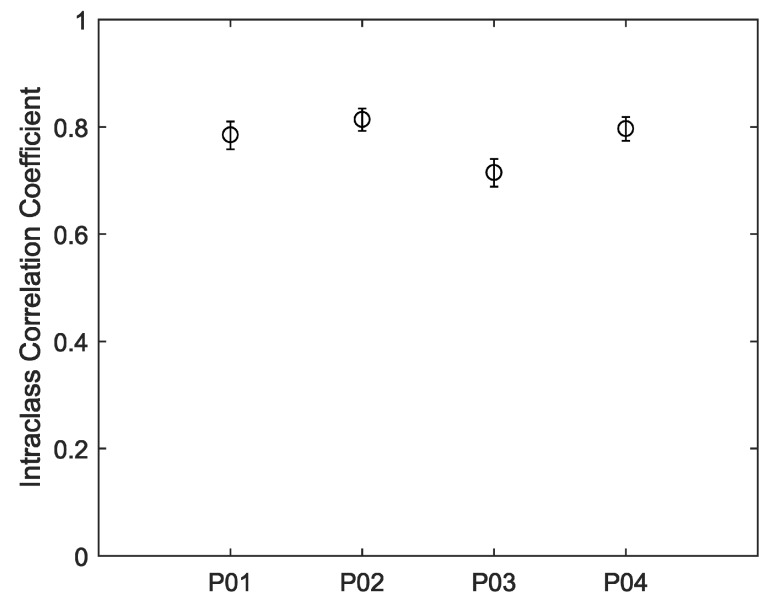
Inter-instrument ICC values for 12 h of everyday living.

The devices were also worn by participants for 12-h periods during uncontrolled everyday living activities. The recorded heart rates are shown in Figure 5. For all four participants, much of the day was spent engaged in relatively sedentary activities corresponding to visibly closer agreements between the devices. Periods of activity correspond to increases in heart rate and visibly decreased agreements between the devices. Intraclass correlations and confidence intervals for treadmill walking and 12-h use are plotted, respectively, in Figure 6 and Figure 7. As anticipated, these indicate poor performance during the treadmill activity. However, as shown in Figure 7, the devices performed more consistently during the prolonged acquisitions of activities of everyday living, when activity levels were generally lower on average.

**Figure 8:**
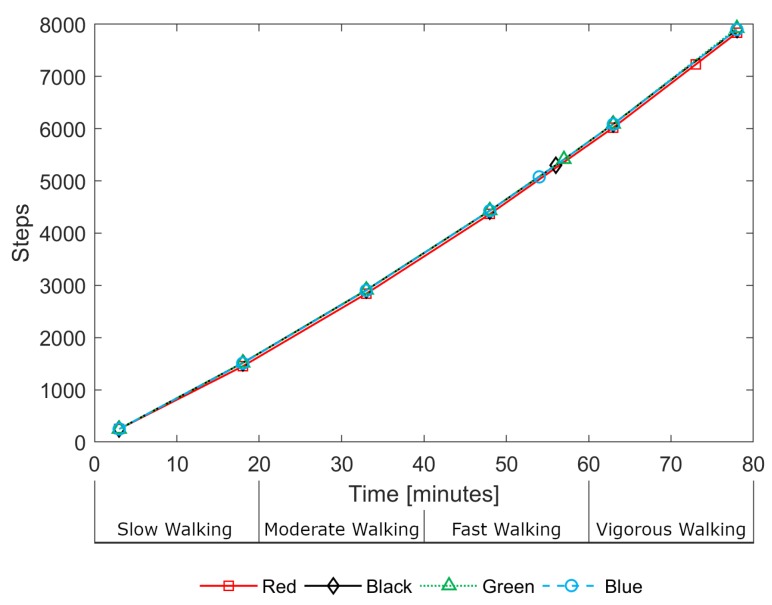
Example of ‘steps’ data (acquisition for participant P03 during treadmill activity).

Step count data was also logged by each device. During the treadmill activity, estimated step counts were in close agreement between devices. Figure 8 illustrates this consistency, showing the step counts for all devices for a single participant (P03) during the treadmill activity. The devices do not record step data as frequently as heart rate estimates, with gaps of up to 15 min between loggings. As a result, there are only a small number of data points over the 80-min treadmill activity. One can observe, in Figure 8, the near-linear relationship between steps and time indicating that, despite the walking speed increasing every 20 min, the number of steps-per-minute remained approximately constant; it is the length of stride that increases rather than the step rate.

**Figure 9:**
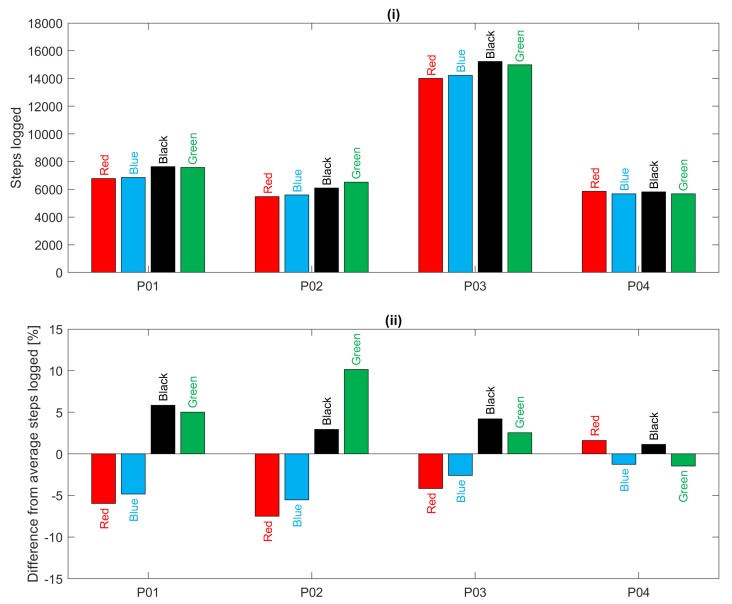
Total steps logged over 12 h of everyday living, (**i**) absolute totals logged by each device for each participant, (**ii**) percentage difference of device totals relative to the average logged step count for each participant.

For the 12-h everyday living recordings, Figure 9 shows a comparison of the total number of steps logged by each device for each participant over the 12-h period. It is clear that participant P03 was the most active during this period. In addition, for all participants, there is good agreement between devices except for a tendency for the devices worn on the left hand (red and blue) to estimate fewer steps than those on the right hand (black and green) as also found in [20,21]. This bias is particularly evident in acquisitions for participants P01, P02 and P03 as illustrated in Figure 9(ii). In summary, during treadmill walking there was poor heart rate agreement between devices but good step count agreement; during everyday living there was better heart rate agreement and slightly deteriorated step count agreement.

## 4. Discussion

As noted earlier, the assessment approach exemplified here is not prescriptive. A useful version assessment approach must incorporate participants and activities that have relevance to the intended study, otherwise, it would have little value. It is also important to ensure that the duration of activities is sufficient for devices to record enough data. We established 20-min durations empirically for each treadmill walking speed by monitoring the frequency of logged heart rate readings and expanding the window to ensure several readings would be logged for each speed.

Of course, a comprehensive assessment using calibration devices (e.g., a 12-lead ECG and a calibrated pedometer) would be preferable to the approach outlined here. Similarly, this empirical approach is preferable to no assessment at all, or reliance on outdated, irrelevant or unreproducible reports in the literature. We can imagine that the sample testing of new and updated devices could provide some reassurance regarding device performance and reliability, and the practice usefully propagated into wearable device applications, for example, applications of sampled wearable heart rate estimates [40] and heart rate variability [41], and across the domain of consumer-level wearable technology research.

Of the several limitations of the presented approach, there was, intentionally, a small number of participants, a limited sample of unrepeated activities and there were no reference recordings for the 12-h everyday living activity. (Reference readings from finger-worn pulse oximeters were attempted, but the devices repeatedly failed to maintain accurate readings). Additionally, the presented approach cannot disambiguate the effects of inter-device variability and variability caused by wearing the device in different positions. However, with just four participants and two activity acquisitions, we were able to quickly and simply obtain an insight into the performance and reliability of the devices *at their current version*, have an appreciation of their limitations and, also, a degree of confidence regarding their potential for study acquisitions.

## 5. Conclusions and Further Work

There is considerable scope for further work to improve reproducibility across the activity monitoring domain and to assist researchers evaluate and re-evaluate new and updated devices. We have demonstrated an empirical approach to device assessment that provides an example assessment that is not onerous and could be repeated without difficulty as and when devices are updated.

Despite issues associated with reliable optical heart rate acquired from the wrist during activity, we might hope that future and updated consumer devices would (i) be better at identifying erroneous values and avoid reporting them and (ii) be better at correctly estimating values. However, it would be unwise to assume every device upgrade will necessarily result in improved device performance in all aspects.

In a systematic review of consumer-wearable activity trackers, Everson et al. [30], recommend that “*future studies on the measurement properties of the trackers should be sure to initialize the tracker properly and indicate in the publication how this was done so others can replicate the process. Providing the specific tracker type, date purchased, and date tested would also be important*”. We additionally recommend that full device details, including software and firmware versions, are reported in the literature. We further recommend that there is some means to enable and encourage the sharing of version-by-version device assessment data between manufacturers, users and researchers.

## Figures and Tables

**Table 1:** Participant Summary.

Participant	Age (Years)	Gender	Height (m)	Weight (kg)	BMI
P01	25	Female	1.69	58	20.03
P02	54	Female	1.62	65	24.7
P03	47	Male	1.75	70	22.8
P04	28	Male	1.70	76	26.2

**Table 2:** The Treadmill Walking Activity Schedule.

Time (Minutes)	20	20	20	20
**Activity**	Slow	Moderate	Fast	Vigorous
	walking	walking	walking	walking
	(2.4 km/h)	(3.2 km/h)	(4.8 km/h)	(6.4 km/h)

**Table 3:** Values of Mean Absolute Percentage Error (MAPE) and IntraClass Correlation (ICC) From Treadmill Walking Activities.

Participant	Black		Blue		Green		Red	
ID	*MAPE*	*ICC*	*MAPE*	*ICC*	*MAPE*	*ICC*	*MAPE*	*ICC*
P01	7.08%	0.68	7.13%	0.71	4.34%	0.81	5.62%	0.90
P02	9.60%	0.69	15.55%	0.67	11.94%	0.58	13.42%	0.71
P03	13.00%	0.47	14.00%	0.02	16.00%	0.19	9.00%	0.84
P04	8.69%	0.84	6.14%	0.91	8.04%	0.86	7.57%	0.89

## Appendix A

The further material details were as follows:

- Garmin Vivosmart 3 software/firmware versions: SW: v4.10; TSC: v1.10; SNS: v5.90. Devices were initialized according to the arm worn and all data was taken directly from logged .FIT files. Devices were purchased on 9 March 2018 and acquisitions made during May 2018. Their serial numbers were as follows: Black—560185378, Red—560185383, Blue—560640435, Green—560639717.
- The treadmill was an h/p/cosmos Pulsar treadmill, h/p/cosmos Sports & Medical Gmbh, Nussdorf-Traunstein, Germany. (cos100420b; ID: X239W80479043; OP19: 0319 1139)
- Polar H10 chest heart rate monitor (FCC ID: INW1W; Model: 1W; IC: 6248A-1W; SN: C7301W0726005; ID: 14C00425; Firmware: 2.1.9 and data acquired via Polar Beat 2.5.3.
